# Supplementary material for: Comparative study on cytogenetics and transcriptome between diploid and autotetraploid rice hybrids harboring double neutral genes
Source: PLoS One. 2020 Sep 28;15(9):e0239377. doi: 10.1371/journal.pone.0239377 (PMC7521696; doi:10.1371/journal.pone.0239377)
Supplement: S1 File — S1 Fig Chromosome behaviors during PMC meiosis in DF1 (diploid hybrid); S2 Fig Chromosome behaviors during PMC meiosis in AF1 (autotetraploid hybrid); S3 Fig Morphological characteristics of F1 hybrid and their parents with different ploidy levels. S4 Fig The correlation coefficient of different ploidy hybrids and their parents in anthers at meiosis and single microspore stages. S5 Fig Confirmation of the DEGs in T449 and autotetraploid F1 hybrid during meiosis stage. S6 Fig Twelve possible additive and nonadditive gene expression patterns in autotetraploid hybrid relative to its parents. (PDF) [file pone.0239377.s001.pdf]

Supporting Information

S1 File. Additional figures about meiosis, plant morphology and gene expression in autotetraploid and diploid rice

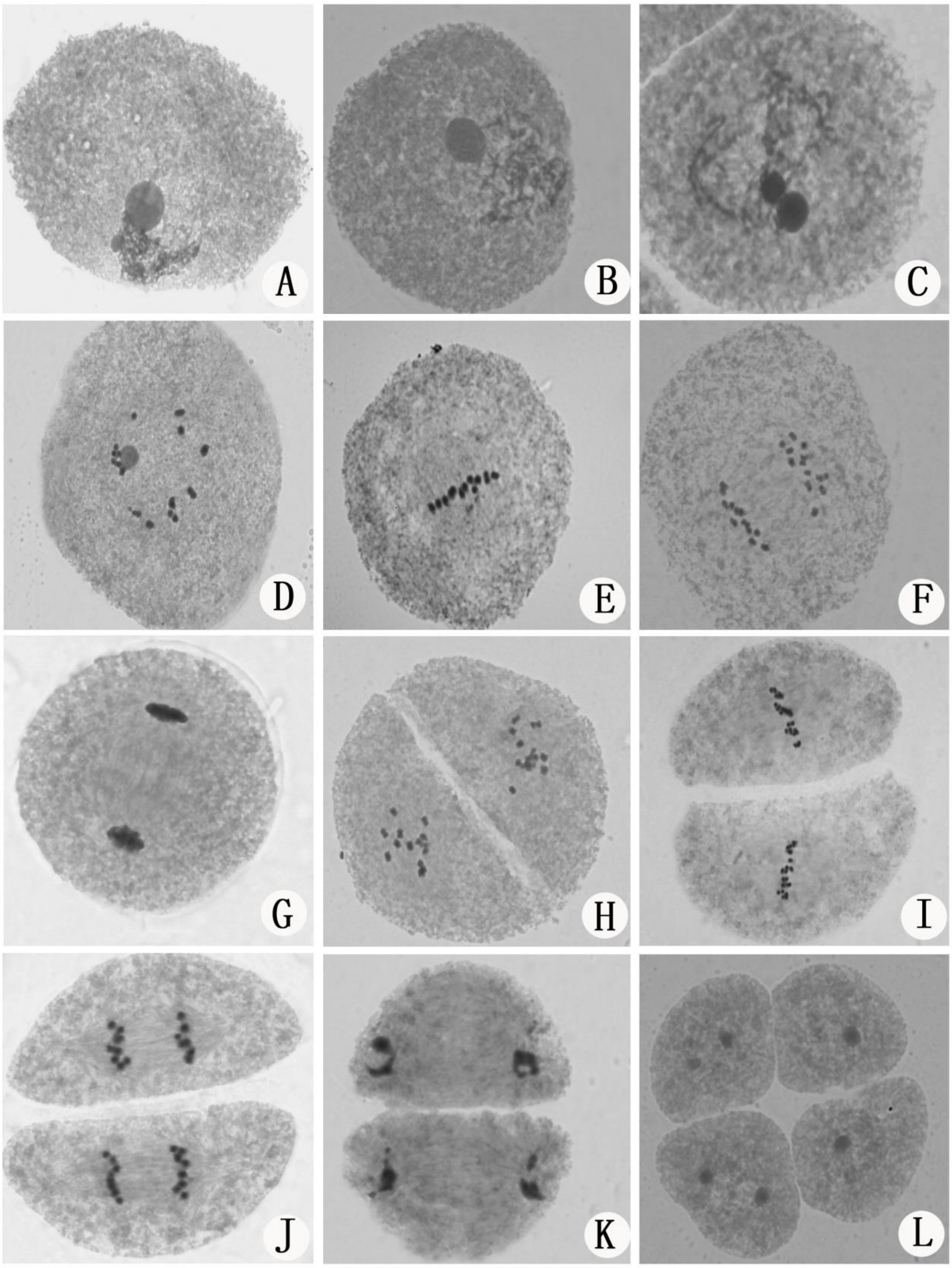

S1 Figure Chromosome behaviors during PMC meiosis in DF_1 (Diploid hybrid)

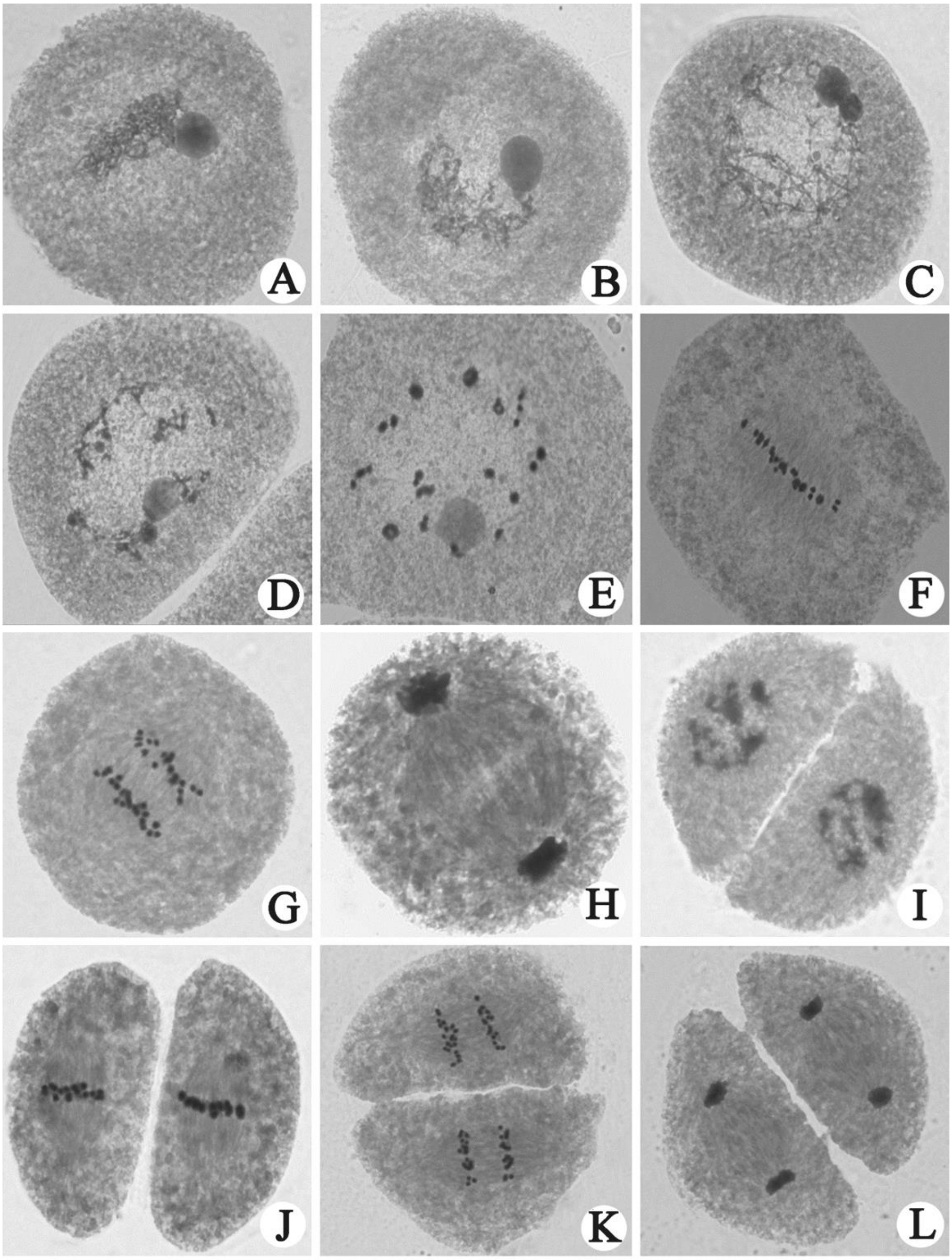

S2 Figure Chromosome behaviors during PMC meiosis in AF_1 (Autotetraploid hybrid)

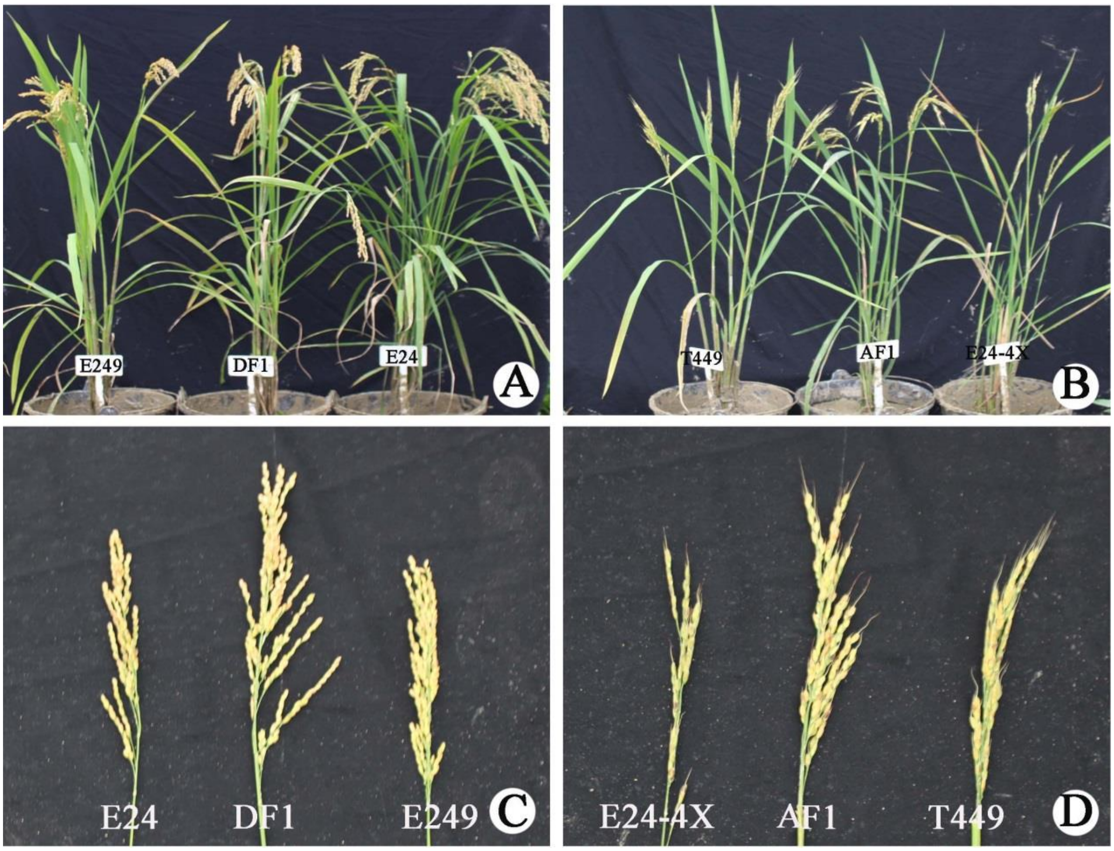

S3 Figure Morphological characteristics of F_1 hybrid and their parents with different ploidy levels.

Note: E24 and E249 indicate diploid rice lines, and DF_1 indicates their F_1 hybrid; E24-4x and T449 indicate autotetraploid rice lines, and AF_1 indicates their F_1 hybrid.

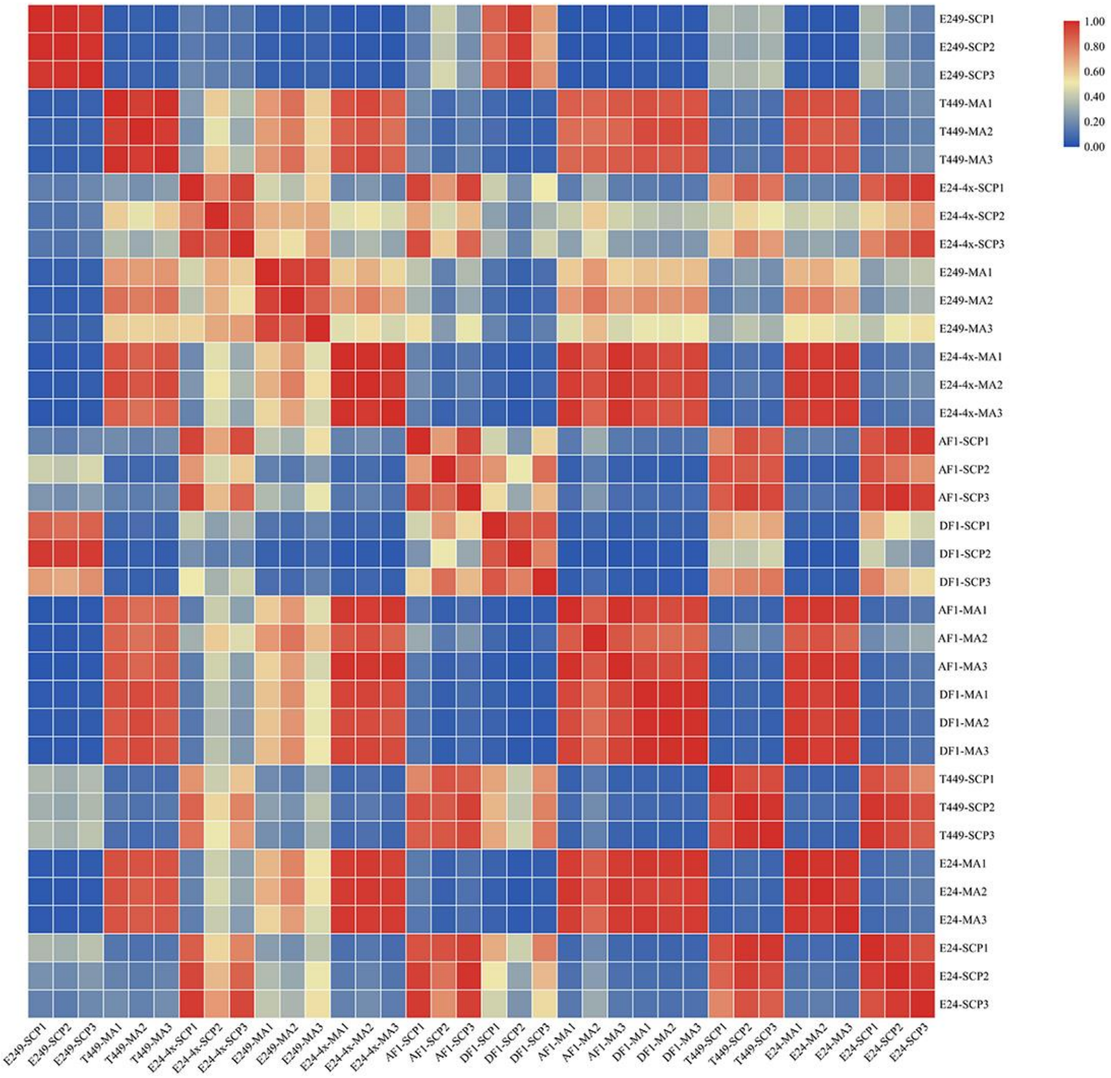

S4 Figure The correlation coefficient of different ploidy hybrids and their parents in anthers at meiosis and single microspore stages

Note: E24 and E249 indicate diploid rice lines, and DF_1 indicates their F_1 hybrid; E24-4x and T449 indicate autotetraploid rice lines, and AF_1 indicates their F_1 hybrid.

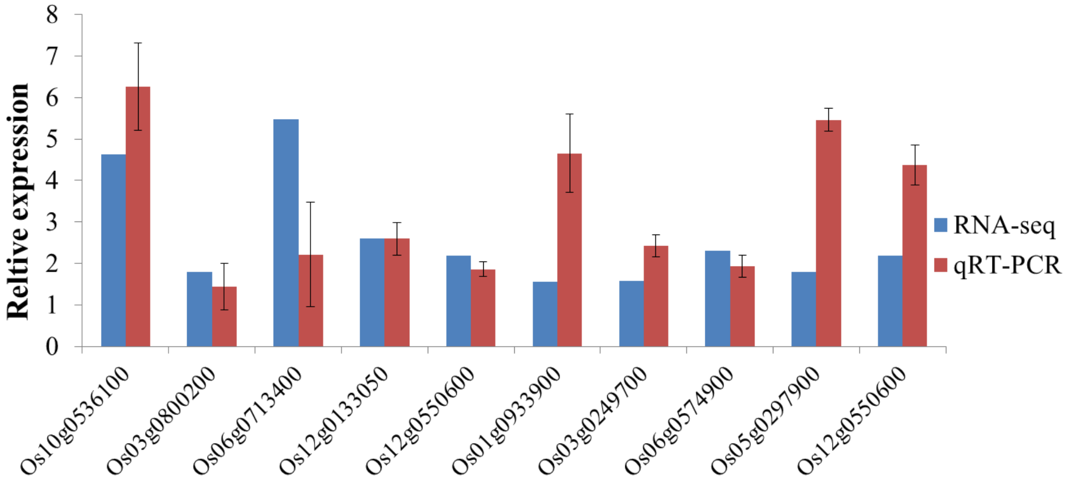

S5 Figure Confirmation of the DEGs in T449 and autotetraploid F_1 hybrid during meiosis stage.

The X- and Y -axis are representing the genes and relative expression levels, respectively. The error bars indicate the standard deviation (SD) of three biological replicates.

Categories	Additivity		Parental expression level dominance				Transgressive down-regulation			Transgressive up-regulation		
			High		Low							
	I	II	III	VI	V	VI	VII	VIII	IX	X	XI	XII
	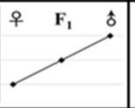	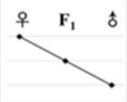	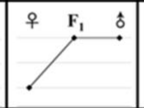	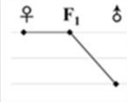	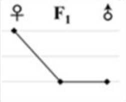	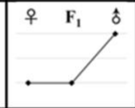	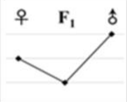	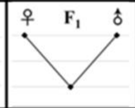	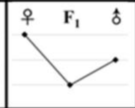	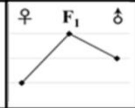	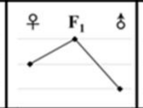	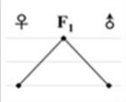
MA	15	9	92	15	189	9	12	124	413	20	1	5
SCP	8	13	15	22	3	1	1	1	0	2	2	0
Common DEGs	1	0	4	2	0	0	0	0	0	0	0	0

S6 Figure Twelve possible additive and nonadditive gene expression patterns in autotetraploid hybrid relative to its parents.

Note: MA and SCP represent meiosis and single microspore stage, respectively.
