# Supplementary material for: Comparative study on cytogenetics and transcriptome between diploid and autotetraploid rice hybrids harboring double neutral genes
Source: PLoS One. 2020 Sep 28;15(9):e0239377. doi: 10.1371/journal.pone.0239377 (PMC7521696; doi:10.1371/journal.pone.0239377)
Supplement: Attachment — Submitted filename: Response to Reviewers-8-10.docx [file pone.0239377.s003.docx]

**Dear Editor**

Thank you very much for taking interest in our research article (PONE-D-20-14708). We are truly grateful to yours and other reviewers’ critical comments and valuable suggestions. We have revised the manuscript according to reviewers' suggestions and have incorporated all the changes suggested by reviewers. As the reviewers have main concern about the interpretation of results and figures, so we have added new table and figures, revised sentences in the results section, and figures and figure legends. In addition, we also have improved the language/spelling mistakes of the revised version. We acknowledge the reviewers for their valuable suggestions that considerably improved the quality and contents of the manuscript. Following is our point-by-point response to the reviewer’s comments:

Reviewers' comments:

Reviewer's Responses to Questions

**Comments to the Author**

1. Is the manuscript technically sound, and do the data support the conclusions?

The manuscript must describe a technically sound piece of scientific research with data that supports the conclusions. Experiments must have been conducted rigorously, with appropriate controls, replication, and sample sizes. The conclusions must be drawn appropriately based on the data presented.

Reviewer #1: Yes

Reviewer #2: Yes

Reviewer #3: Yes

**Response:** All the reviewers agreed with the quality of manuscript and we have further revised the manuscript carefully based on the suggestions of three reviewers.

2. Has the statistical analysis been performed appropriately and rigorously?

Reviewer #1: Yes

Reviewer #2: Yes

Reviewer #3: Yes

**Response:** All the reviewers agreed the statistical analysis performed in the manuscript. Thanks

3. Have the authors made all data underlying the findings in their manuscript fully available?

The [PLOS Data policy](http://www.plosone.org/static/policies.action#sharing) requires authors to make all data underlying the findings described in their manuscript fully available without restriction, with rare exception (please refer to the Data Availability Statement in the manuscript PDF file). The data should be provided as part of the manuscript or its supporting information, or deposited to a public repository. For example, in addition to summary statistics, the data points behind means, medians and variance measures should be available. If there are restrictions on publicly sharing data—e.g. participant privacy or use of data from a third party—those must be specified.

Reviewer #1: Yes

Reviewer #2: Yes

Reviewer #3: Yes

**Response:** All the reviewers are agreed that manuscript adhere to the PLOS Data Policy.

4. Is the manuscript presented in an intelligible fashion and written in standard English?

PLOS ONE does not copyedit accepted manuscripts, so the language in submitted articles must be clear, correct, and unambiguous. Any typographical or grammatical errors should be corrected at revision, so please note any specific errors here.

Reviewer #1: Yes

Reviewer #2: Yes

Reviewer #3: Yes

**Response:** All the reviewers are agreed that manuscript is presented in an intelligible fashion and written in standard English.

5. Review Comments to the Author

Please use the space provided to explain your answers to the questions above. You may also include additional comments for the author, including concerns about dual publication, research ethics, or publication ethics. (Please upload your review as an attachment if it exceeds 20,000 characters)

**Reviewer #1**: In this paper Chen et al., 2020, compared cytological and transcriptome data between autotetraploid and diploid rice hybrid during meiosis and single microspore stages to understand the molecular mechanism of neutral genes for overcoming pollen sterility in autotetraploid rice hybrids. Using cytological analysis they identified lower chromosomal abnormalities in autotetraploid compared to other lines. The authors provide a theoretical basis for molecular mechanisms of heterosis in autotetraploid rice.
I would suggest to improve the quality of the figures and have some minor comments for the paper:

**Response:** We are very thankful for reviewer for encouraging comments about our manuscript and we have tried our best to improve the manuscript as suggested by reviewer.

- Page 5, Line 2-3, which lines were crossed?

**Response:** We have revised the sentence as follow.

We developed autotetraploid hybrid by crossing T449 with E24-4x, diploid rice hybrids by crossing E249 with E24-2x.

- Page 8, in the first paragraph, authors needs to describe the general design of experiment, which lines they crossed and which diploid and tetraploid lines they developed for analysis. Also authors needs to explain what they are showing in the figure.

**Response:** Thank you for useful suggestion, and we have revised the first paragraph as follow:

In this study, T449 and E249, harboring *S_a_^n^* and *S_b_^n^* double neutral genes for pollen sterility loci, were used to develop autotetraploid rice hybrid (hereafter referred as TF1) and diploid rice hybrid (hereafter referred as DF1) by crossing with E24-4x and E24-2x. There were different alleles at three pollen sterility loci (i.e. Sa, Sb, and Sc) in diploid and autotetraploid parents, so these hybrids presented allelic "interactions" at three pollen sterility loci. In diploid hybrid, pollen fertility was 68.67%, which was lower than its parents, and mid parent heterosis (MPH) and high parent heterosis (HPH) of pollen fertility were -24.17% and -24.98%, respectively. But in autotetraploid hybrid, pollen fertility was 76.74%, which was similar to their parents, and MPH and HPH of pollen fertility were 4.43% and 1.59%, respectively (Table1 and Figure 1). We detected high pollen fertility (>65%) in different ploidy hybrids with double neutral genes at Sa and Sb pollen sterility loci, and these results showed that different ploidy hybrids had no interaction at Sa and Sb loci. In addition, the MPH and HPH of autotetraploid hybrid pollen were higher than diploid hybrid, which showed that the effect of double neutral genes were stronger in autotetraploid hybrid.

- The nomenclature of hybrids used in Figure 2 is different than what is described at Page 8, line 19-21

**Response**: Sorry, the figure 2 is wrong, and we have replaced it with a new figure.

Fig. 2 Frequency of normal cells in different ploidy hybrids during PMCs meiosis

- Page 9, Line 19-20, authors needs to describe at least some of the genes validated by qRT-PCR

**Response:** Thanks for useful suggestion, and we have revised it.

In total, ten genes were randomly selected from RNA-seq data to validate by qRT-PCR, including MADS gene (MADS56, *Os10g0536100*), meiosis related-gene (*Os03g0800200*), F-box gene (*Os06g0713400*), 60S acidic ribosomal protein (*Os12g0133050*), glutathione S-transferase (*Os01g0933900*), signal Peptidase homologue (*Os05g0297900*), two hypothetical proteins (*Os12g0550600* and *Os12g0550600*) and two expressed protein (*Os03g0249700* and *Os06g0574900*). The qRT-PCR results were similar to the RNA-seq data (S5 Figure), which proved that the RNA-seq data is dependable.

- Page 10, Line 20, please explain what these results show?

**Response:** We revised the subtitle and paragraph.

“Association of DEGs_HP_ with QTLs for yield-related traits”.

We mapped DEGsHP detected in autotetraploid hybrid, which were specifically expressed significant in autotetraploid at the meiosis, to identify QTLs associated with grain yield in the rice genome (http://www.gramene.org). We identified a large number of QTLs for yield-related traits, including grain weight per plant, pollen fertility, spikelet number, 1000-grain weight and male fertility restoration. A total of 22 genes were mapped in the interval of 12 yield-related QTLs, including 1 grain number QTL, 1 pollen fertility QTL, 1 spikelet number QTL, 5 1000-grain weight QTLs and 4 male fertility restoration QTLs. Furthermore, 3, 2, 1,9 and 7 genes were identified in the grain yield per panicle QTLs, pollen fertility QTLs, spikelet number QTLs, 1000-grain weight QTLs and male fertility restoration QTLs, respectively (Table 4). The results of above showed that several differentially expressed genes may be related to autotetraploid hybrid-related traits.

- Similarly Page 12, Line 17, what you want to show by these results?

**Response:** We want to compare the effect of double neutral genes between autotetraploid hybrid and diploid hybrid. We have revised it. Thanks for useful suggestion.

**Reviewer #2:** The manuscript entitled “Comparative study on cytogenetics and transcriptome between diploid and autotetraploid rice hybrids harboring double neutral genes” is focused on the role of neutral genes in diploid and autotetraploid rice. The mechanism of pollen sterility is very complex and hot issues for rice breeders. Neutral genes are great source to overcome reproductive isolation in indica-japonica hybrids. The results indicated that meiosis-specific and meiosis-related genes, and amino acids and photosynthesis metabolism-related genes contribute to higher yield and pollen fertility in autotetraploid rice hybrid. Overall, the manuscript is well written and executed but there are few issues which should be resolved before final acceptance.

**Response**: We are very thankful for reviewer for encouraging comments about our manuscript and we have tried our best to improve the figures and results section as suggested by reviewer.

Major Concern

Authors have published an article about double neural genes previously (Wu et al. 2017; Rice). What are major differences between these studies. If any data similar, should cite a reference. How about data submission to NCBI?

**Response:** Wu et al. (2017) showed that double pollen fertility neutral genes could overcome hybrids sterility caused by the multi-pollen sterility loci interactions in autotetraploid rice hybrids, but this study was planned to observe phenotype and molecular mechanism of parents and hybrids with double neutral genes in different ploidy rice. This study contains no published data and all cytological/morphological observations done in this sudy, and we have added following statement in the manuscript. All the data has been submitted to NCBI (Accession IDs in Genbank: PRJNA656120, and transcriptome data of T449 and E249 was downloaded from NCBI under the accession number PRJNA436888 [23].

Minor concerns
P7L21, P8L1-5, please delete/re-write from the results section.

**Response:** Thanks for your suggestion, we revised the sentence.

In this study, T449 and E249, harboring *S_a_^n^* and *S_b_^n^* double neutral genes for pollen sterility loci, were used to develop autotetraploid rice hybrid (hereafter referred as TF1) and diploid rice hybrid (hereafter referred as DF1) by crossing with E24-4x and E24-2x, these hybrids have different pollen sterility loci interactions at *Sa, Sb*, and *Sc* loci.

P8 Authors could add more cytological results, such as images about abnormal chromosome behavior in diploid and autotetraploid rice.

**Response:** Thanks for your suggestion. We have added the following table about cytological results.

Table S2 Number of observed cells and frequency of normal cells in different ploidy hybrids

Figure 1 legends need to improve.

**Response:** We have added more detail in the figure legend as follow:

“A, E249 (Diploid parent harboring double neutral genes); B, DF_1_ (F_1_ diploid hybrid); C, E24 (Diploid Parent); D, T449 (Autotetraploid parent harboring double neutral genes); E, AF_1_ (F_1_ Autotetraploid hybrid); F, E24-4X (Autotetraploid parent). Round and fully stained pollens represent fertile pollens (green arrows), while other represent abnormal pollens (white arrows). Bar=100um.”.

**Reviewer #3:** The authors try to clarify the role of double neutral genes associated with pollen fertility in autotetraploid rice hybrid; however, all results are on the way to the goal and far from the conclusions that the authors claimed in the Discussion. There are several mistakes in the manuscript that authors need to properly addressed. I have following concerns about the manuscript:

**Response**: We are very thankful for reviewer for useful suggestions about our manuscript and we have tried our best to revise the manuscript as suggested by reviewer.

Introduction
Page 5 line 2-4: ‘we developed autotetraploid and diploid rice hybrids by crossing T449, …’ Are autotetraploid and diploid rice all crossing T449? I think this is a wrong sentence.

**Response:** Sorry there is a mistake in expression of material. We have revised it.

In this study, we developed autotetraploid rice hybrids by crossing T449 with E24-4x, diploid rice hybrids by crossing E249 with E24-2x, which contained double neutral genes, *S_a_^n^* and *S_b_^n^*, to evaluate the effect of double neutral genes caused by polyploidy.

Methods
Page 6 line 22. gene Ontology should be Gene Ontology
**Response:** Thanks for your suggestion, we have revised it.

Results
Page 8 line 1-7: This paragraph is almost the introduction.

**Response:** Thanks for your suggestion, we revised this paragraph.

In this study, T449 and E249, harboring *S_a_^n^* and *S_b_^n^* double neutral genes for pollen sterility loci, were used to develop autotetraploid rice hybrid (hereafter referred as TF1) and diploid rice hybrid (hereafter referred as DF1) by crossing with E24-4x and E24-2x, respectively. There were different alleles at three pollen sterility loci (i.e. *Sa*, *Sb*, and *Sc*) in diploid and autotetraploid parents, so these hybrids presented allelic "interactions" at three pollen sterility loci. In diploid hybrid, pollen fertility was 68.67%, which was lower than its parents, and mid parent heterosis (MPH) and high parent heterosis (HPH) of pollen fertility were -24.17% and -24.98%, respectively. But in autotetraploid hybrid, pollen fertility was 76.74%, which was similar to their parents, and MPH and HPH of pollen fertility were 4.43% and 1.59%, respectively (Table1 and Figure 1). We detected high pollen fertility (>65%) in different ploidy hybrids with double neutral genes at *Sa* and *Sb* pollen sterility loci, and these results showed that different ploidy hybrids had no interaction at *Sa* and *Sb* loci. In addition, the MPH and HPH of autotetraploid hybrid pollen were higher than diploid hybrid, which showed that the effect of double neutral genes were stronger in autotetraploid hybrid.

Page 8 line 8-9: Please re-phrase.

**Response:** There are different alleles at three pollen sterility loci (i.e. *Sa*, *Sb*, and *Sc*) in diploid and autotetraploid parents, and hybrids presented allelic "interactions" at these three loci.

Page 8 line 11: I cannot see the difference in pollen fertility between the materials in figure 1 clearly. The seed set of the materials should be present in the manuscript.
**Response:** We added the arrows in figure 1, it is showed that what pollen is normal or abnormal. The seed set of the materials are presented in S2 Table.

Figure 1 Pollen fertility of hybrids and parents with different ploidy levels.

A, E249 (Diploid parent harboring double neutral genes); B, DF1 (F1 diploid hybrid); C, E24 (Diploid Parent); D, T449 (Autotetraploid parent harboring double neutral genes); E, TF1 (F1 Autotetraploid hybrid); F, E24-4X (Autotetraploid parent). Round and fully stained pollens represent fertile pollens. Bar=100um. White and green arrow present abnormal and normal pollen, respectively.

Page 8 line 15-21: How many PMCs at each stage during meiosis the authors observed? Why are there no figures of the abnormal chromosome behavior in DF1 and TF1?
**Response:** Sorry, the figure 2 is wrong. Six materials were observed during meiosis, E249, E24, DF1, T449, E24-4x and TF1. We have replaced it with a new figure and add a table about number of PMCs. This table contain the number of cells observed, along with other data.

Fig. 2 Frequency of normal cells in different ploidy hybrids during PMCs meiosis

Page 8 line 19: “…was lower than its parents in the metaphase I, …” I could only found T449 in the figure 2, where are the data of T431, T435 and T438 during meiosis?
**Response:** Sorry, this is a mistake, the figure 2 is wrong, and we have replaced it with new figure.

Table S2 Number of observed cells and frequency of abnormal cells in different ploidy hybrids

Fig. 2 Frequency of normal cells in different ploidy hybrids during PMCs meiosis

Page 9 line 2: Lower pollen fertility of diploid hybrid (~70%) was found, the seed set of diploid hybrid was similar to the parents (90%). The pollen fertility of TF1 was similar to DF1, but the seed set is lower than diploid hybrid. How to explain it? What’s the connection of Double neutral genes in TF1 and DF1? What’s the purpose of this analysis? To evaluate the heterosis of autotetraploid hybrid and diploid hybrid, or ploidy analysis between TF1 and DF1, or double neutral genes analysis, it makes me very confusing.
**Response:** The mechanism of the lower seed set of autotetraploid rice hybrid is rather complex. The abnormalities in the fertilization and meiosis process might lead to low seed set except pollen fertility. Double pollen fertility neutral genes can improve pollen fertility of autotetraploid and diploid rice hybrid. But the pollen fertility of TF1 is higher than their patents. The purpose of this research is to understand the molecular mechanism of neutral genes for overcoming pollen sterility in autotetraploid rice hybrids.

Page 9 line 17: the table S4 should be present in the figure.
**Response:** Thanks for your useful suggestion and we have added a new figures in the manuscript.

S4 Figure The correlation coefficient of different ploidy hybrids and their parents in anthers at meiotic and single microspore stages

Page 9 line 18: Figure S4, the authors randomly selected 10 genes for qPCR assay, why all these genes showed up-regulation. How about the down-regulated genes?
**Response:** We didn’t select the down-regulated genes, and these 10 genes were randomly that showed up-regulation. We randomly selected 10 genes to confirm our RNA-seq data, but we selected 10 genes is all up-regulation by accidentally.

Discussion
The discussion is not so well written. How to get the conclusion of the first subhead in the discussion? Is this only base on hybrid vigour? “But the frequency of chromosome behavior of the diploid rice hybrids carrying San and Sbn neutral genes and its parents were similar” How to get this result? Where are the statistical results of this part? I CAN’T FIND IN THE RESULTS SECTION.

**Response:** Thanks for a critical comment. Because the figure 2 is wrong, so statement was not in accordance with the results presented in previous version of manuscript. We have replaced an old figure with a new figure.  The chromosome behavior of diploid rice hybrid and its parents was similar, and their abnormal frequency of chromosome behavior was lower than 10% during meiotic stage. Figure 2 is clearly demonstrating the difference in chromosome configuration between diploid and autotetraploid hybrid and these results support our conclusion about the effect of double neutral genes in polyploid rice.

*Again, we appreciate all of your insightful comments. Thank you for taking your time and energy to help us to improve the manuscript.* *We hope that the revised version of our manuscript is now suitable for publication in PLOSONE.*

Sincerely,

MQ Shahid (on behalf of all co-authors)

Associate Professor

College of Agriculture

South China Agricultural University

Guangzhou, 510642, P.R. China
