# Supplementary material for: Comparative study on cytogenetics and transcriptome between diploid and autotetraploid rice hybrids harboring double neutral genes
Source: PLoS One. 2020 Sep 28;15(9):e0239377. doi: 10.1371/journal.pone.0239377 (PMC7521696; doi:10.1371/journal.pone.0239377)
Supplement: Attachment — Submitted filename: Response to Reviewers 2020-08-28.docx [file pone.0239377.s004.docx]

**Dear Editor**

Thank you very much for taking interest in our research article (PONE-D-20-14708R1). We are truly grateful to yours and other reviewers’ critical comments and valuable suggestions. We have revised the manuscript according to reviewers' suggestions and have incorporated all the changes suggested by reviewer. We acknowledge the reviewers for their valuable suggestions that considerably improved the quality and contents of the manuscript. Following is our point-by-point response to the reviewer’s comments:

Reviewers' comments:

Reviewer's Responses to Questions

**Comments to the Author**

1. If the authors have adequately addressed your comments raised in a previous round of review and you feel that this manuscript is now acceptable for publication, you may indicate that here to bypass the “Comments to the Author” section, enter your conflict of interest statement in the “Confidential to Editor” section, and submit your "Accept" recommendation.

Reviewer #1: (No Response)

Reviewer #2: All comments have been addressed

Reviewer #3: All comments have been addressed

**Response:** We are very thankful to reviewers for encouraging comments about our manuscript.

2. Is the manuscript technically sound, and do the data support the conclusions?

The manuscript must describe a technically sound piece of scientific research with data that supports the conclusions. Experiments must have been conducted rigorously, with appropriate controls, replication, and sample sizes. The conclusions must be drawn appropriately based on the data presented.

Reviewer #1: (No Response)

Reviewer #2: Yes

Reviewer #3: Yes

**Response:** The reviewers agreed with the quality of manuscript and we have further revised the manuscript carefully based on the reviewer suggestions.

3. Has the statistical analysis been performed appropriately and rigorously?

Reviewer #1: (No Response)

Reviewer #2: Yes

Reviewer #3: Yes

**Response:** The reviewers agreed with the statistical analysis performed in the manuscript. Thanks

4. Have the authors made all data underlying the findings in their manuscript fully available?

The [PLOS Data policy](http://www.plosone.org/static/policies.action#sharing) requires authors to make all data underlying the findings described in their manuscript fully available without restriction, with rare exception (please refer to the Data Availability Statement in the manuscript PDF file). The data should be provided as part of the manuscript or its supporting information, or deposited to a public repository. For example, in addition to summary statistics, the data points behind means, medians and variance measures should be available. If there are restrictions on publicly sharing data—e.g. participant privacy or use of data from a third party—those must be specified.

Reviewer #1: (No Response)

Reviewer #2: Yes

Reviewer #3: Yes

**Response:** The reviewers are agreed that manuscript adhere to the PLOS Data Policy.

5. Is the manuscript presented in an intelligible fashion and written in standard English?

PLOS ONE does not copyedit accepted manuscripts, so the language in submitted articles must be clear, correct, and unambiguous. Any typographical or grammatical errors should be corrected at revision, so please note any specific errors here.

Reviewer #1: (No Response)

Reviewer #2: Yes

Reviewer #3: Yes

**Response:** The reviewers are agreed that manuscript is presented in an intelligible fashion and written in standard English.

6. Review Comments to the Author

Please use the space provided to explain your answers to the questions above. You may also include additional comments for the author, including concerns about dual publication, research ethics, or publication ethics. (Please upload your review as an attachment if it exceeds 20,000 characters)

Reviewer #1: (No Response)

Reviewer #2: (No Response)

Reviewer #3: The authors have revised the manuscript carefully according to reviewers’ suggestion. The authors have added new table and figures, revised sentences in the results section, and figures and figure legends. So, readers can follow the contents easily and obtain very useful information from the paper. This manuscript could be accepted for publication after being incorporated few minor suggestions as follow.

**Response:** We are very thankful to reviewer for encouraging comments about our manuscript and we have tried our best to improve the manuscript as suggested by reviewer.

1. In Plant material: It is mentioned that “E249, and its autotetraploid rice line, T449, harboring San and Sbn”. The authors need to write the origin of these materials in detail.

**Response:** We have mentioned the origin of these cultivars in M&M section. Thanks

2. Page 27. Bar=100um maybe Bar=100µm.

**Response:** Thanks for indicaitng a mistake in figure legends, and we have revised it according to reviewer suggestion.

3. Please cross check all the references carefully.

**Response:** We have carefully revsied references in the manuscript.

4. Please be consistent with line name, E24-2x could be change as E24

**Response:** Thanks for useful suggestion and we have mentioned it as E24 in whole manuscript.

*Again, we appreciate all of your insightful comments. Thank you for taking your time and energy to help us to improve the manuscript.* *We hope that the revised version of our manuscript is now suitable for publication in PLOSONE.*

Sincerely,

MQ Shahid (on behalf of all co-authors)

Associate Professor

College of Agriculture

South China Agricultural University

Guangzhou, 510642, P.R. China
